# Force Myography for Monitoring Grasping in Individuals with Stroke with Mild to Moderate Upper-Extremity Impairments: A Preliminary Investigation in a Controlled Environment

**DOI:** 10.3389/fbioe.2017.00042

**Published:** 2017-07-27

**Authors:** Gautam P. Sadarangani, Xianta Jiang, Lisa A. Simpson, Janice J. Eng, Carlo Menon

**Affiliations:** ^1^MENRVA Research Group, School of Engineering Science, Simon Fraser University, Burnaby, BC, Canada; ^2^Graduate Program in Rehabilitation Sciences, University of British Columbia, Vancouver, BC, Canada; ^3^Rehabilitation Research Program, GF Strong Rehab Centre, Vancouver Coastal Health Research Institute, Vancouver, BC, Canada; ^4^Department of Physical Therapy, University of British Columbia, Vancouver, BC, Canada

**Keywords:** activity monitoring, force myography, stroke rehabilitation, grasp detection, wearable sensors

## Abstract

There is increasing research interest in technologies that can detect grasping, to encourage functional use of the hand as part of daily living, and thus promote upper-extremity motor recovery in individuals with stroke. Force myography (FMG) has been shown to be effective for providing biofeedback to improve fine motor function in structured rehabilitation settings, involving isolated repetitions of a single grasp type, elicited at a predictable time, without upper-extremity movements. The use of FMG, with machine learning techniques, to detect and distinguish between grasping and no grasping, continues to be an active area of research, in healthy individuals. The feasibility of classifying FMG for grasp detection in populations with upper-extremity impairments, in the presence of upper-extremity movements, as would be expected in daily living, has yet to be established. We explore the feasibility of FMG for this application by establishing and comparing (1) FMG-based grasp detection accuracy and (2) the amount of training data necessary for accurate grasp classification, in individuals with stroke and healthy individuals. FMG data were collected using a flexible forearm band, embedded with six force-sensitive resistors (FSRs). Eight participants with stroke, with mild to moderate upper-extremity impairments, and eight healthy participants performed 20 repetitions of three tasks that involved reaching, grasping, and moving an object in different planes of movement. A validation sensor was placed on the object to label data as corresponding to a grasp or no grasp. Grasp detection performance was evaluated using linear and non-linear classifiers. The effect of training set size on classification accuracy was also determined. FMG-based grasp detection demonstrated high accuracy of 92.2% (σ = 3.5%) for participants with stroke and 96.0% (σ = 1.6%) for healthy volunteers using a support vector machine (SVM). The use of a training set that was 50% the size of the testing set resulted in 91.7% (σ = 3.9%) accuracy for participants with stroke and 95.6% (σ = 1.6%) for healthy participants. These promising results indicate that FMG may be feasible for monitoring grasping, in the presence of upper-extremity movements, in individuals with stroke with mild to moderate upper-extremity impairments.

## Introduction

Stroke is one of the most prevalent causes of adult disability (Gresham et al., 2004; World Health Organization, 2006). Individuals with stroke often experience impairments in the upper extremity, including a reduction in fine motor control, that contribute to difficulties in completing activities of daily living (ADL), such as dressing, feeding, and home management (Gresham et al., 2004). There is increasing evidence that it is necessary to practice hundreds, if not thousands, of grasp and release motions to optimize hand motor recovery after stroke (Nudo et al., 1996; Murata et al., 2008). As it is unlikely that traditional stroke rehabilitation services can accommodate one-on-one therapies for many hours of practice, this has resulted in increased demand for the creation of wearable sensors to assist therapists and patients in monitoring the large number of repetitions needed to promote motor recovery. Such wearable sensing technologies could be used outside of the clinic to monitor “homework,” as task practice outside of structured therapy sessions can improve neurological recovery and functional abilities (Dobkin, 2004; Harris et al., 2009). Wearable sensors could also be used to encourage individuals with stroke to continue to use their affected limb as part of ADL and therefore avoid the “learned non-use” phenomenon (Taub et al., 2006).

Several types of wearable solutions have been proposed for upper-extremity activity monitoring. One type of sensing system utilizes wrist-worn accelerometers. With appropriate signal processing, accelerometer-based devices have been shown to provide metrics that correlate to the amount of upper-extremity movement achieved by individuals with stroke (Uswatte et al., 2000, 2005, 2006) and the amount of hand use in older adults (Rand and Eng, 2010). However, such devices are unable to determine if the user has successfully grasped an object and are thus unable to distinguish functional use of the upper extremity from non-functional movements, such as shaking or swinging of the arm. Given that the ultimate goal of the rehabilitation process is to restore, enhance, and maintain functional ability (Granger, 1998), it is desirable for rehabilitation sensing devices to be able to distinguish between functional and non-functional movement. To detect functional movement, an activity monitor would need to be able to detect both upper-extremity movement and functional use of the hand.

The use of magnetic sensing systems for detecting wrist and hand movements has also been proposed (Friedman et al., 2014). Such devices use a wrist-worn magnetometer to detect relative movement of a magnetic object donned on a different body part, such as a finger. Friedman et al. have successfully demonstrated the feasibility of such devices at detecting the joint movements associated with the wrist and finger (Rowe et al., 2013; Friedman et al., 2014). However, magnetic sensing systems are susceptible to magnetic interference from other ferromagnetic materials, such as household electronics and hence may have difficulty accurately monitoring functional activity in the home environment (Friedman et al., 2014). Several gloves embedded with sensors for tracking hand postures have also been developed and successfully evaluated (Dipetro et al., 2008). However, such devices can be challenging to don on individuals with limited range of motion and spasticity. In addition, the donning of gloves results in a reduction in palmar sensation and may hamper the completion of fine-motor tasks.

Surface electromyography (sEMG) has been successfully used to detect hand activities in individuals with stroke (Lee et al., 2011; Zhang and Zhou, 2012; Li et al., 2014). Lee et al. (2011) used sEMG data from individuals with stroke to identify six different hand postures. Despite these promising results, challenges remain in the use of sEMG for unobtrusive monitoring of hand motion. sEMG requires the use of bulky electrodes with complex signal acquisition and amplification hardware, and a high signal sampling rate. In addition, given that sEMG is measuring the electrical activity of muscles, its signal-to-noise ratio is sensitive to the positioning of electrodes as well as the skin impedance (Merletti et al., 2001), which can be affected by the presence of hair, sweat, and skin creams. Mechanomyography (MMG) is a complimentary sensing technology, which involves transducing the mechanical oscillations of muscle fibers as they are recruited (Islam et al., 2013). MMG senses the mechanical analog of the motor unit electrical activity measured by sEMG (Islam et al., 2013) and has been shown to be capable of detecting the functional state of the hand using pattern recognition and classification techniques (Islam et al., 2013). However, the performance of MMG is similarly sensitive to sensor placement, and sensor pressure (i.e., adherence) on the surface of the skin (Islam et al., 2013), and requires similarly complex signal-processing methodologies, as those used in sEMG classification.

Force myography (FMG) is an alternative technique that involves monitoring the force, or pressure, at the surface of the limb, as a means to characterize the state of the underlying musculotendinous complex (Wininger et al., 2008). FMG provides a simple-to-use, inexpensive, and unobtrusive method for sensing muscle activity. In contrast to electromyography, the FMG signal can be extracted using off-the-shelf force-sensing elements and does not require complex signal-processing circuitry (Dementyev and Paradiso, 2014). In addition, FMG does not require the sensors to be placed at specific anatomical points on the body to ensure adequate signal acquisition (Castellini et al., 2014), as is required in sEMG (Merletti et al., 2001) and MMG (Islam et al., 2013). Given these considerations, a growing amount of research is exploring the use of FMG for activity-tracking, gesture-recognition, and the detection of functional tasks. FMG systems have been used to predict grip strength (Wininger et al., 2008) and single-finger forces (Castellini and Ravindra, 2014). More recently, FMG has been combined with machine learning and pattern recognition techniques to create systems that can detect upper-extremity postures (Xiao and Menon, 2014), hand gestures (Dementyev and Paradiso, 2014), and repetitions of grasp and move tasks in healthy participants (Sadarangani and Menon, 2015). Dementyev et al. demonstrated classification accuracy in excess of 80% when classifying six different grasp types using an FMG band with healthy participants (Dementyev and Paradiso, 2014). Li et al. (2012) demonstrated classification accuracy of 99% at classifying 17 different grasp types using a high-density FMG array with healthy participants. The results of these studies give credence to the concept of classifying the FMG signal to detect grasping.

Despite these promising results, little work has been done to establish the feasibility of using FMG for detecting grasping in populations with upper-extremity impairments, who might ultimately benefit from this technology. Yungher and Craelius (2012) have shown that FMG is an effective method for providing biofeedback to improve fine motor function, as part of structured upper-extremity rehabilitation for individuals with upper-extremity impairment, including individuals with stroke and individuals with traumatic brain injury. In their study, the authors requested participants to pinch at predetermined intervals and used the difference between FMG signatures for the requested pinch gesture and a template pinch gesture to provide biofeedback to participants. Experimental results indicate that gesture recognition-based biofeedback improved the outcomes of repetitive task training. While the results of this study give credence to the concept of using FMG sensing for providing biofeedback in rehabilitation applications, the study did not evaluate the utility of FMG for monitoring and distinguishing between grasping and no grasping, in the presence of other upper-extremity movement which the FMG signal has also been shown to be sensitive to Xiao and Menon (2014). Instead, the proposed system involved monitoring the FMG signature of a single grasp type, which was elicited at a known time, in the absence of any other upper-extremity movement. To be suitable for monitoring functional use of the hand in daily living, for rehabilitation applications, FMG sensing systems will need to be able to detect grasping in individuals with stroke, without knowing when the grasp may be elicited, despite the presence of other confounding upper-extremity movement.

Several characteristics associated with individuals with stroke may impact the feasibility of using FMG for detecting grasping in this population. Individuals with stroke have reduced muscular strength (Bohannona, 1987) and greater muscle spasticity (Watkins et al., 2002) which may affect the magnitude and quality of the FMG signal. In addition, movements completed with the stroke-affected limb have reduced range of motion, are less smooth, and involve variations in speed and acceleration when compared to the less affected limb (Kamper et al., 2002). Given that the FMG signal is sensitive to postures of the hand, wrist, forearm, and elbow (Xiao and Menon, 2014), the movement features of the stroke-affected limb, and the associated compensatory mechanisms employed, could introduce challenges when detecting grasping in the presence of movement in the three-dimensional workspace. Furthermore, for FMG to be feasible in busy clinical settings, the initial setup of the device (i.e., number of grasp repetitions required to train the grasp detection classifier) would need to be minimal in time and in repetitions.

This study explores the feasibility of using FMG, with machine learning techniques, for grasp detection in the presence of upper-extremity movements, for individuals with stroke who have arm and hand impairments. As noted, the use of FMG classification for grasp detection continues to be an active area of research in healthy volunteers. However, given that the FMG signal also captures information related to upper-extremity movements and postures other than grasping, the use of FMG classification for grasp detection in individuals with stroke, who may have altered muscular characteristics and movement patterns, has yet to be established. Feasibility was assessed by (1) establishing and comparing the accuracies of FMG-based grasp detection for individuals with stroke and healthy individuals, with FMG data corresponding to grasping, releasing, moving while grasping, and moving without grasping, using linear and non-linear classifiers and (2) determining and comparing the amount of training data necessary to accurately classify a grasp with participants with stroke and healthy participants. We considered the device to be feasible if the grasp detection accuracy was at least 90%, and if the training data required for the classifier were at most 50% of the size of the testing data. We hypothesized that (1) the use of FMG for grasp detection in individuals with stroke would be feasible, yielding an accuracy of at least 90% with training data that are at most 50% of the size of testing data, in a controlled environment, (2) classification accuracy would be lower for participants with stroke when compared to healthy participants, and (3) more data would be required for accurate grasp detection for participants with stroke when compared to healthy participants.

## Materials and Methods

### Participants

Eight participants with stroke and eight healthy participants were recruited for this study. Inclusion criteria for the participants with stroke were as follows: (1) cerebrovascular accident confirmed by MRI or CT scan and (2) mild to moderate impairment of the paretic hand, confirmed by Chedoke Hand Score >5 (Gowland et al., 1993), and poorer performance on the Box and Blocks test (Mathiowetz et al., 1985) for the paretic hand compared to the non-paretic hand. Healthy participants were a sample of convenience of right-dominant adults with no history of injuries or impairments to their upper limbs.

Participants with stroke and healthy participants had a mean age of 69 years (σ = 6 years) and 27 years (σ = 7 years), respectively. All participants with stroke were chronic stroke survivors (>12 months poststroke). The Chedoke Arm Score was a mean of 6.75 (σ = 0.71), and the Chedoke Hand Score was a mean of 6.38 (σ = 0.75), both with a range of 5–7 for all participants with stroke. Paretic side performance was 62.1% (σ = 22.3%) of the non-paretic side performance for the Box and Blocks Test (Mathiowetz et al., 1985).

### Experimental Setup

**Figure 1:**
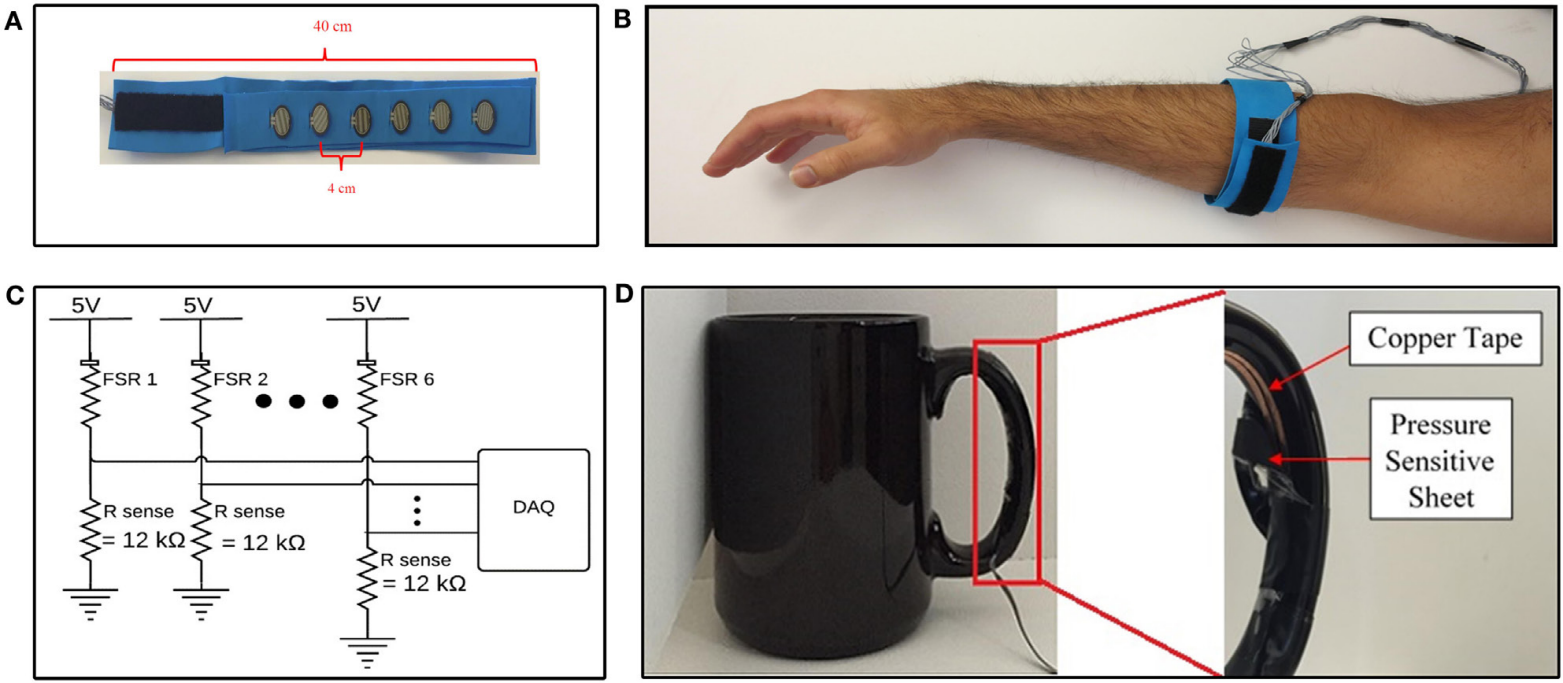
Data acquisition devices and systems. **(A)** Internal view of force-sensing band. **(B)** Force-sensing band donned on forearm. **(C)** Data acquisition system. **(D)** Internal view of validation sensor on cup handle.

Six commercial off-the-shelf force-sensitive resistors (FSRs) (model number FSR 402) manufactured by Interlink Electronics (2015) were embedded into a 40 cm flexible foam band, to form a force-sensing band to collect FMG data from participants (Figure 1A). Foam was chosen as the material for the band due to its conformability and flexibility, which would ensure adherence of the band to the working limb of the participant, and would be comfortable to don. The number of sensors was chosen as a trade-off between collecting more channels of data, and limiting the stiffness of the band, as each sensing element contributed to the stiffness of the band. The number of sensors selected for this study is similar to the quantity of sensors that have been shown to be sufficient for accurate FMG acquisition, from healthy volunteers, in other studies (three to eight sensors) (Wininger et al., 2008; Xiao and Menon, 2014; Sadarangani and Menon, 2015). The sensors were placed 4 cm apart from each other, to create a total sensing area of approximately 20 cm. An additional 4 cm of space was left on either side of the sensor array. The remaining 12 cm of the band was covered with Velcro^®^, to enable fastening of the band. The band was positioned around the participant’s working forearm, approximately 8 cm from the olecranon, to detect FMG signals associated with the functional state of the participant’s working hand (Figure 1B). The band was fastened with the aforementioned Velcro^®^ such that the band was tight, and could not slip or rotate, but was still comfortable for each participant. None of the participants reported discomfort due to band tightness. This method of controlling for tightness was adopted from the literature, as it has been shown to produce high FMG classification accuracies in healthy volunteers (Li et al., 2012; Xiao and Menon, 2014). To emulate the intended use condition, only the distance from the olecranon was specified, and the rotational placement of the band on forearm musculature (e.g., anterior or posterior) was not specified. The device was donned on the impaired side (paretic side) for participants with stroke and donned on the dominant side for healthy individuals.

The FMG signal was quantified from the FSRs using a voltage divider circuit (Figure 1C). The sense resistor, *R*_sense_, in the voltage divider circuit controls the sensitivity of the FSR and represents a trade-off between the force-sensing range (i.e., maximum force the can be measured before saturation), and full scale output range (i.e., maximum output signal at maximum sensing range) of the sensing circuit (Interlink Electronics, 2015). The sense resistor was empirically chosen to be 12 kΩ for the selected donning position. The voltage across the sense resistors was sampled between the 0 and 5 V range, with a 12-bit resolution, at 20 Hz, using a National Instruments DAQ Device (National Instruments, 2016) interfaced with custom LabVIEW software. The 20 Hz sampling rate was selected as it has been shown to be sufficient to achieve high FMG classification accuracies in the literature (Radmand et al., 2016). The resulting data acquisition system is schematically depicted in Figure 1C.

A coffee cup, weighing 530 g, was selected as the object for all grasp and move tasks in the experimental protocol. The cup was instrumented with a validation sensor (Figure 1D) to detect when the cup had been grasped. The validation sensor comprised of a pressure-sensitive conductive sheet (3M, 2017b) attached to conductive copper tape (3M, 2017a). The pressure-sensitive conductive sheet, which demonstrates a reduction in resistance as the applied force increases, was wrapped around the inside of the cup handle. Copper tape was attached to the two ends of the sheet and interfaced to the data acquisition system, to quantify the resistance displayed by the pressure-sensitive conductive sheet. The pressure-sensitive conductive sheet and copper tape on the cup handle were wrapped in insulation tape to ensure no other source of conductance or resistance was electrically connected to the copper tape. Data from this sensor were acquired using the same data acquisition system that was used for FMG data (Figure 1C). Data from this sensor were used to label FMG data as corresponding to a grasp or neutral hand posture and to calculate grasp detection accuracy. If the signal from the validation sensor was above a configurable voltage, the sensor was considered to have detected a grasp. The experimenter visually confirmed that the validation sensor was accurately detecting grasping throughout the data collection session using readouts on the custom LabVIEW software. The experimenter also observed the study to ensure that no objects other than the instrumented cup were grasped by the participant throughout the duration of the experimental protocol and that the participants always picked up the cup using the instrumented handle. The validation sensor, with the insulation tape peeled away to reveal the copper tape and pressure-sensitive conductive sheet, is depicted in Figure 1D.

### Experimental Protocol

**Figure 2:**
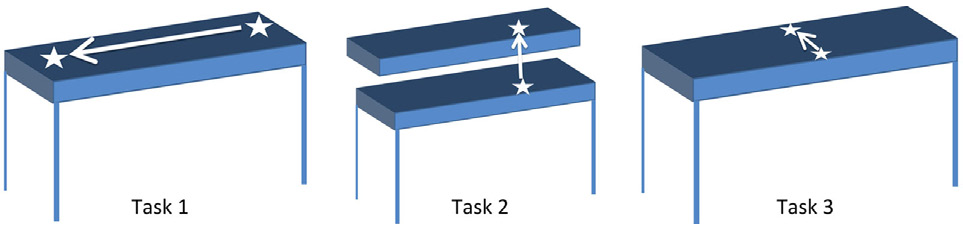
Start and end positions for Tasks 1–3. White arrow indicates direction of motion from start to end position for each task.

Participants were seated comfortably in an armless chair with their working hand resting on the table in front of them. They were asked to reach for, grasp (i.e., pick up) and move a cup across three different planes of movement, resulting in three different tasks (Figure 2). For each task, participants were asked to reach for the cup at the start position, grasp the cup, lift it off the table, move it to the end position, place the cup down on the table at the end position, and completely release the cup. Subsequently, they were asked to reach for the cup at the end position, grasp the cup, lift it off the table, move it to the start position, place the cup down on the table at the start position, and completely release the cup before returning their hands to rest on the table in front of them. In Task 1, the end position was to the left of the start position, such that participants moved the cup laterally. In Task 2, the end position was the top of a custom shelf, above the start position, such that participants lifted the cup upwards. In Task 3, the end position was ahead of the start position, such that participants moved the cup forward. The target distance for each task was set to approximately 90% of the maximum active range of motion each participant was comfortably able to achieve. The participants were asked to repeat each task, by first moving the cup from the start position to the end position, and subsequently moving the cup from the end position back to the start position, 10 times at a comfortable pace. This resulted in 20 grasp and move actions per task.

The objective of this study was to evaluate the feasibility of using FMG classification for detecting grasping, despite the variety of hand positions and upper-extremity movements that individuals may use when completing tasks in daily living. Hence, participants were free to move their shoulder, elbow, and wrist positions in whichever manner they found suitable while moving the cup to and from the target locations. In addition, participants were asked to grasp the cup using the instrumented handle, but were free to use any grasp type that would allow them to do so, such as the medium wrap or abducted thumb grasps (Cutkosky, 1989). Furthermore, participants were free to determine the specific orientation of their fingers around the cup’s handle. FMG-based grasp detection with grasp and move tasks was tested as these tasks are a common activity used for upper limb training post stroke (Pollock et al., 2013). In addition, the described protocol enabled evaluating FMG classification accuracy for grasp vs. neutral hand posture (i.e., no grasp), with data corresponding to grasping, releasing, moving while grasping, and moving without grasping, in the three planes of movement within which we perform our daily activities. Thus, to achieve accurate grasp detection, the FMG classifier would need to be able to detect the FMG signal patterns associated with a grasp despite variations in grasp types used, and despite the presence of confounding upper-extremity movements, and hand positions, that also affect FMG signal patterns.

### FMG Grasp Detection (Classification) Accuracy Analysis

Feasibility of FMG grasp detection among individuals with stroke was assessed by examining and comparing grasp classification accuracy and the amount of training data necessary for accurate grasp classification for participants with stroke and healthy participants. As noted previously, we considered FMG-based grasp detection to be feasible if the grasp detection accuracy was at least 90%, and if the training data required for the classifier were at most 50% of the size of the testing data.

**Figure 3:**
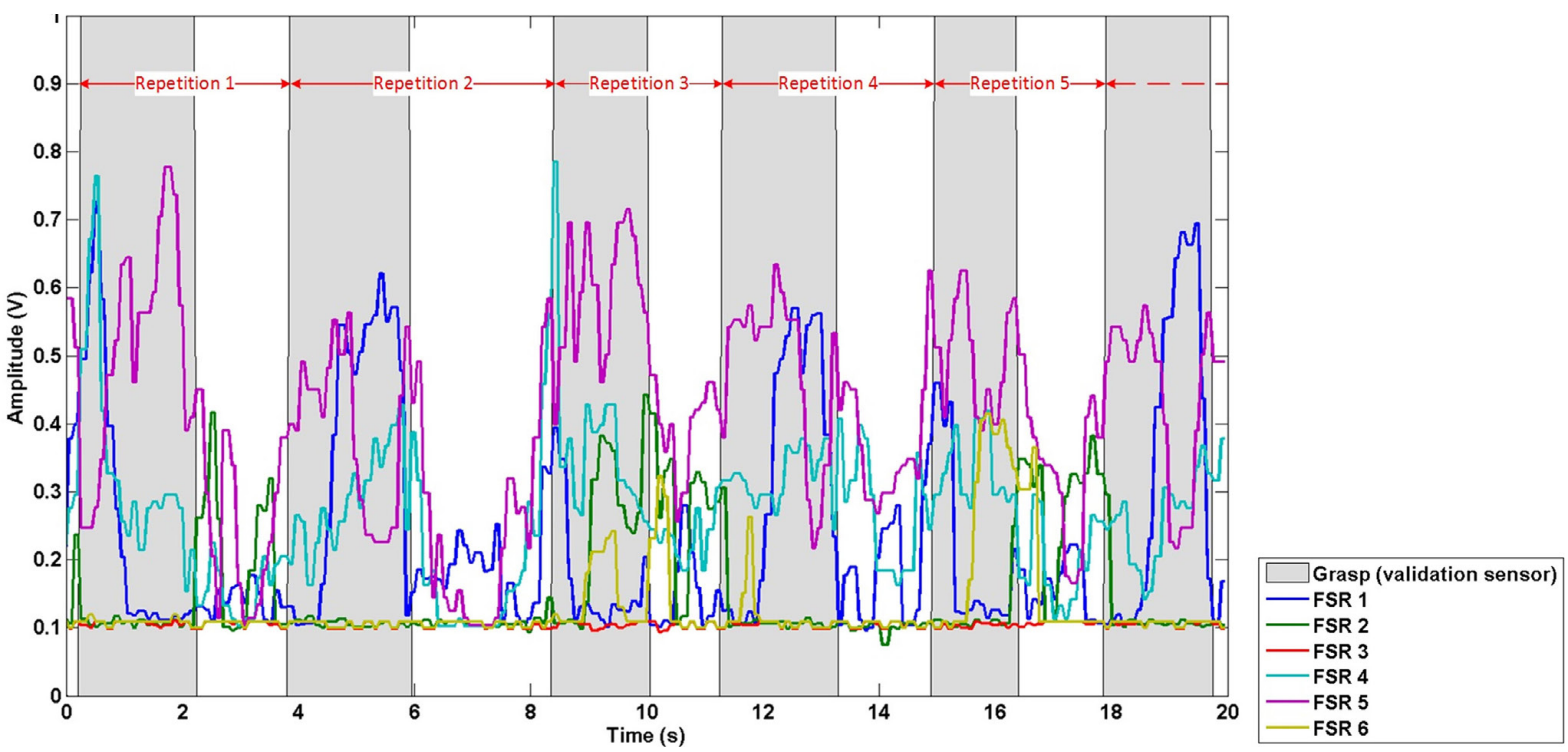
Exemplary repetition division scheme. Red arrows demark the division of data into repetitions based on the signal from the validation sensor (gray shading).

Data were first temporally segmented into repetitions, based on the onset of grasps derived from the validation sensor output, to allow for evaluation of the effect of training set size on classification accuracy. Data from the start of a grasp to the start of the next grasp were assigned to one repetition, based on amplitude threshold crossings from the validation sensor. Hence, each repetition comprised of FMG data (from all six FSRs) corresponding to the grasp and move action required to move the cup, and the subsequent hand and arm activity corresponding to reaching and other preparatory movement leading up to the next grasp and move action. Data corresponding to the reaching and other preparatory movement in-between grasps were included to establish the classifiers robustness to false-positives (i.e., incorrectly predicting a grasp when the participant had a neutral hand posture). The repetition division scheme is depicted in Figure 3, where points of time labeled as grasp (grasp and move action) by the validation sensor are shaded in gray, points of time labeled as neutral hand posture (reaching and other preparatory movement) are not shaded. The FMG data in the figure are divided into repetitions (red arrows) based on the labels of the validation sensor (gray shading).

A two-fold cross-validation scheme was employed. Each data set related to a task for a participant (20 repetitions) was divided into training and testing sets. In the first fold, data from the 1st to 10th repetitions formed the training set and the remaining 10 repetitions formed the testing set. In the second fold, the data from the 11th to 20th repetition were used to form the training set and the first 10 repetitions formed the testing set. Classification accuracy was then computed using the average classification accuracy obtained across the two-folds. Before classification, data within the training set and testing set were filtered using a median filter. The window size for the median filter was empirically selected to be three samples (300 ms). Subsequently, both the training and testing data were normalized with respect to the maximum and minimum values within the training data set. The training set was then used for generating a model for each task for each subject, and the testing set was used for model evaluation. Classification accuracy was then calculated as the average accuracy seen across the two-folds.

For the purpose of accuracy analysis, classification output was categorized into true positive (TP), true negative (TN), false positive (FP), and false negative (FN). A TP corresponds to the classifier correctly predicting a grasp when the participant was grasping the cup, as labeled by the validation sensor. A TN corresponds to the classifier correctly predicting a neutral hand posture when the participant had a neutral hand posture (during reaching and preparatory motion), as labeled by the validation sensor. An FP corresponds to the classifier incorrectly predicting a grasp when the participant had a neutral hand posture, as labeled by the validation sensor. An FN corresponds to the classifier incorrectly predicting a neutral hand posture when the participant was grasping the cup, as labeled by the validation sensor. Grasp detection accuracy was then computed as per Eq. 1.

(1) $$\text{accuracy}(\%)=\frac{\text{TP}+\text{TN}}{\text{TP}+\text{TN}+\text{FP}+\text{FN}}$$

For example, a typical participant took approximately 4 s to complete a repetition (i.e., a grasp and move action, and the subsequent reaching and preparatory motion leading up to the next grasp and move action), resulting in 80 instantaneous samples per channel (sampled at 20 Hz). Of these 80 samples, approximately 60% (48) of the samples would correspond to points of time when the participant was grasping and moving the cup, as labeled by the validation sensor. The remaining 40% (32) of the samples would correspond to points of time when the participant had a neutral hand gesture. During these points of time the participant could have been reaching for the cup, engaging in other preparatory motion, or returning to the neutral position. A 100% accuracy would indicate that the classifier was able to correctly identify the aforementioned 48 grasp samples as grasp, and the remaining 32 neutral samples as neutral hand posture.

Classification was carried out using a support vector machine (SVM) classifier with a non-linear radial basis function (RBF) (Bishop, 2006) kernel given by the following expression (Eq. 2):

(2) $$K(x_{i},x_{j})=e^{\left(-\gamma ||x_{i}-x_{j}{||}^{2}\right)}$$

where *x_i_* and *x_j_* are two arbitrary samples from the input data, and $||x_{i}-x_{j}{||}^{2}$ is the squared Euclidean distance between the two specific samples, γ is the symbol for gamma, a parameter used to control the fitting behavior of the SVM. The LIBSVM library was used off-line in the MATLAB environment to evaluate the accuracy of the RBF-SVM, with default cost and gamma parameters (Chang and Lin, 2011). To evaluate the ease of separation of FMG data, the use of a linear discriminant analysis (LDA) classifier was also evaluated using the MATLAB Statistics and Machine Learning Toolbox (MathWorks, 2015). The LDA assumes that the input data are normally distributed. The Kolmogorov–Smirnov test was used to verify this assumption with the acquired data before classification was conducted. Unlike the RBF-SVM that seeks a linear separator in the non-linear feature space, the LDA seeks a linear decision boundary in the data space (Bishop, 2006). The ability to use simpler linear signal-processing and classification methods would indicate that an FMG-based grasp detection system could potentially be more easily embedded into a compact and portable, low-power device that is capable of running independently.

Classification accuracy was established for each task using the RBF-SVM and LDA classifiers for both healthy participants and individuals with stroke. A two-way ANOVA was conducted to examine the effect of the two independent variables: (1) participant type (individuals with stroke vs. healthy individuals) and (2) classifier type (RBF-SVM vs. LDA), on the dependent variable of classification accuracy. The significance level was set to 0.05.

To examine the effect of training data size on classification accuracy, we varied the size of the training set from one repetition to 10 repetitions for each fold within the two-fold cross-validation scheme. In all cases, accuracy was determined by classifying data within the fixed-sized testing set, comprising data associated with 10 repetitions. The training set size required to achieve the target 90% average accuracy across the two-folds was established for participants with stroke and healthy participants for both types of classifiers. The correlation coefficient between training set size and classification accuracy was also derived for participants with stroke and healthy participants.

## Results

All eight participants with stroke and eight healthy participants successfully completed the data collection protocol. The Kolmogorov–Smirnov test confirmed that the acquired data were normally distributed, and hence suitable for classification using LDA.

**Figure 4:**
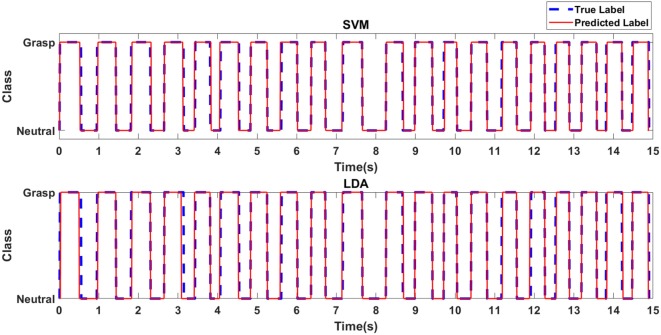
Predicted class label in relation to true class label for exemplary case of Task 1 for the first participant with stroke.

Figure 4 depicts the predicted class label in relation to the true class label for the RBF-SVM and LDA classifiers for an exemplary case of Task 1 for the first participant with stroke. Table 1 shows the accuracies associated with grasp detection for each task, for participants with stroke and healthy participants using the RBF-SVM and LDA.

**Table 1:** Average classification accuracy for participants with stroke, and healthy participants, for each task using the RBF-SVM and LDA classifiers.

Task	Participants with stroke				Healthy participants			
	Support vector machine (SVM)		Linear discriminant analysis (LDA)		SVM		LDA	
			
	Accuracy (%)	σ (%)	Accuracy (%)	σ (%)	Accuracy (%)	σ (%)	Accuracy (%)	σ (%)
Task 1	93.6	2.1	93.4	2.0	97.1	1.3	96.7	1.6
Task 2	91.4	4.6	89.9	5.0	95.1	2.5	93.6	3.9
Task 3	91.7	5.7	91.4	6.2	95.6	2.5	94.4	3.1

*Task 1, Task 2, and Task 3 are the grasp and move tasks in the lateral, upward, and forward directions, respectively*.

Grasp detection accuracy was lower for participants with stroke, when compared to healthy volunteers. Average grasp detection accuracy was 92.2% (σ = 3.5%) and 91.6% (σ = 3.8%) across all tasks for participants with stroke using the RBF-SVM and LDA, respectively. Average grasp detection accuracy was 96.0% (σ = 1.6%) and 94.9% (σ = 2.3%) across all tasks for healthy participants using the RBF-SVM and LDA, respectively. The two-way ANOVA revealed that the higher classification accuracies obtained for healthy individuals when compared to individuals with stroke was statistically significant (*F*_2,31_ = 11.65, *p* < 0.005). There was no significant difference in classification performance between the RBF-SVM and LDA classifiers, and there was no interaction effect between participant type and classifier type.

Classification accuracies obtained were lower for moving upwards (Task 2), or forwards (Task 3), when compared to moving laterally (Task 1) for both participants with stroke and healthy participants. To explore the possibility of using a single model to detect grasping across all movement directions, a single classifier model was constructed using data from all three tasks. In specific 50% of the repetitions from all three tasks were used for training, and 50% of the repetitions from all three task were used for testing. A two-fold cross-validation scheme was employed. The classification accuracies obtained were lower than those obtained when using a single classifier model for each task. Classification accuracies for participants with stroke were 91.6% (σ = 3.4%) and 90.9% (σ = 3.4%) using the RBF-SVM and LDA classifiers, respectively. The classification accuracies obtained for healthy participants were 94.9% (σ = 2.5%) and 94.0% (σ = 2.8%), using the RBF-SVM and LDA classifiers, respectively.

**Figure 5:**
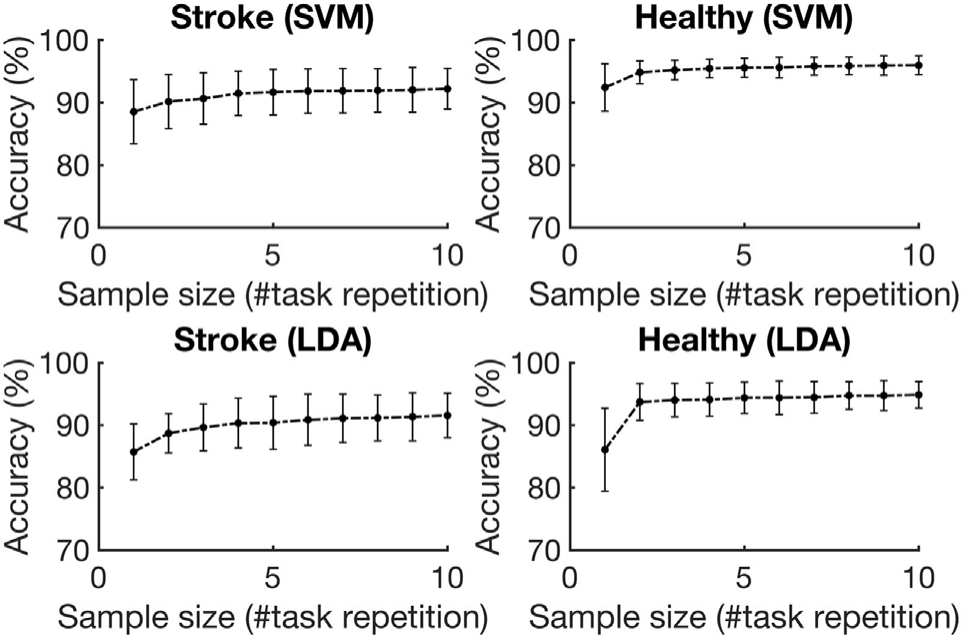
Average classification accuracy vs. training set size. The accuracies were averaged across the three tasks and eight subjects for each population, for training set sizes ranging from 1 repetition to 10 repetitions. The error bars represent 1 SD.

**Figure 6:**
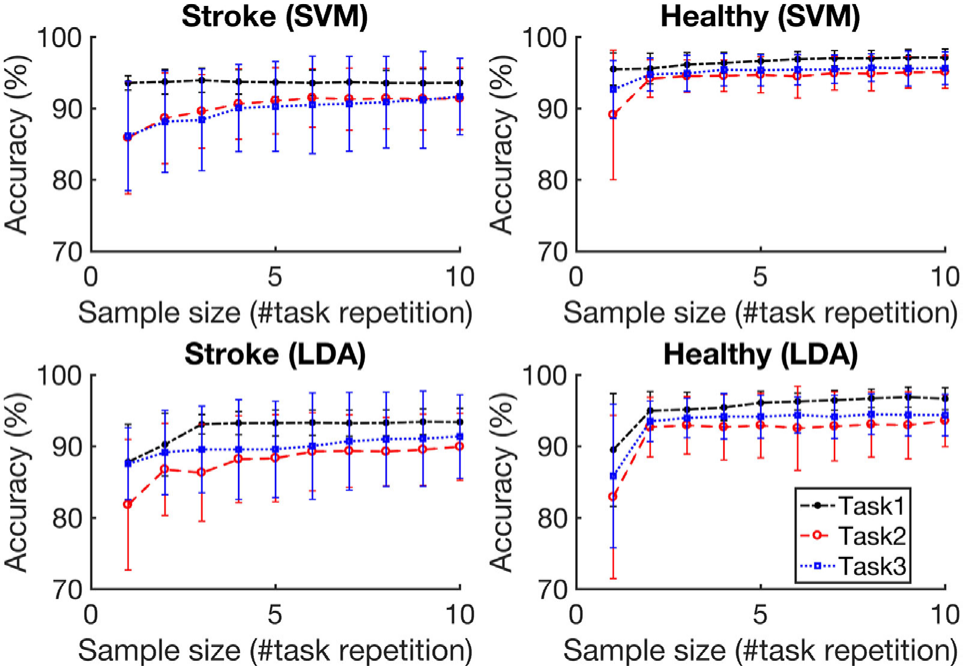
Classification accuracy vs. training set size for each task. Task 1, Task 2, and Task 3 are the grasp and move tasks in the lateral, upward, and forward directions, respectively. The accuracies were averaged across eight subjects for each population. The error bars represent 1 SD.

Figure 5 depicts the average accuracies taken across both folds and all tasks, for training sets of differing sizes for participants with stroke and healthy participants. The accuracies associated with each task are depicted in Figure 6. The accuracy was dependent on the size of the training set provided. The correlation coefficients between the number of training samples and the average accuracy for participants with stroke are 0.8606 (*p* = 0.001) using the RBF-SVM, and 0.8488 (*p* = 0.002) using the LDA. The correlation coefficients between the number of training samples and the accuracy obtained for healthy participants are 0.7604 (*p* = 0.011) using the RBF-SVM, and 0.6254 (*p* = 0.05) using the LDA. The use of two and four repetitions was necessary for achieving greater than 90% accuracy for participants with stroke using the RBF-SVM and LDA, respectively (Figure 5). The use of one and two repetitions was sufficient for achieving greater than 90% accuracy for healthy participants using the RBF-SVM and LDA, respectively (Figure 5).

## Discussion

The overall objective of this study was to explore the feasibility of using FMG for grasp detection in individuals with stroke. Grasp detection accuracy was evaluated using an experimental protocol that involved grasping, releasing, moving while grasping, and moving without grasping, in the three planes of movement within which we perform our daily activities. Additional variability was introduced by the variety of movement trajectories that were employed by participants to achieve the tasks, as participants were free to employ varying wrist, elbow, and shoulder motions to complete the target movement required for each task. In addition, participants were also able to vary the grasp type used, as long as they picked up the cup using the instrumented handle. Variability was also introduced into the paradigm by the functional impairments that the participants with stroke demonstrated, which are reflected in the clinical scores. For example, a Chedoke Hand Score of 5 would represent an individual who has difficulty extending their wrist and spreading the fingers apart. Grasp classification accuracy was established and compared for participants with stroke and healthy participants using both RBF-SVM and LDA classifiers. The susceptibility of classification accuracy to the amount of training data provided was also established.

Grasp detection accuracy was lower for participants with stroke when compared to healthy participants. A potential explanation for the lower grasp detection accuracy is the empirically observed variability in grasp types, movement trajectories, and additional compensatory mechanisms demonstrated by participants with stroke. This variability may have resulted in a more challenging classification problem. In addition, participants with stroke often had difficulty in grasping and completely releasing the cup at the start and end positions, which potentially made classification of those points of time more challenging. It is noteworthy that there is an age difference in the groups of participants enrolled in this study. Aging can affect the properties of muscle fibers (Deschenes, 2004) and subcutaneous fat, which may impact the quality of muscle based signals (Vaillancourt et al., 2003). Hence, the difference in accuracies observed between the participants with stroke and healthy individuals may not be related only to the existence of upper-extremity impairment due to stroke. Instead, these results show the accuracy obtainable, within the confines of the experimental protocol, with FMG-based grasp detection for the target population of individuals with stroke, in comparison to the population of healthy, young individuals, in which FMG has been, and continues to be, widely studied (Dementyev and Paradiso, 2014; Xiao and Menon, 2014; Sadarangani and Menon, 2015).

The use of a linear classification scheme did result in lower classification performance for both participants with stroke and healthy participants. However, the differences in classification performance were not statistically significant. Average grasp detection accuracy with the LDA remained above 90% for both participants with stroke and healthy participants, suggesting that linear classification may be adequate for FMG-based grasp detection.

It is noteworthy that classification accuracies were lower for moving upwards (Task 2), or forwards (Task 3), when compared to moving laterally (Task 1) for both participants with stroke and healthy participants. Furthermore, the classification accuracies obtained when using a single classifier model for all three tasks were lower than those obtained when using a single classifier for each task. These variations in accuracy suggest that FMG grasp classification accuracy may be sensitive to movement direction and upper-extremity postures. Despite the noted reductions, average grasp classification accuracy remained above 90% for this combined model, suggesting that the use of a single training round for predicting grasping in all three directions may be feasible.

Overall, FMG-based grasp detection demonstrated high accuracy of 92.2% (σ = 3.5%) with participants with stroke. These promising results met our first feasibility criteria and suggest that the use of FMG for grasp detection in individuals with stroke, in the presence of upper-extremity movement, may be feasible.

Grasp detection accuracy was significantly dependent on training set size in all cases. However, a larger training set size was required to achieve 90% accuracy with participants with stroke when compared to healthy participants. It is possible that a larger training set size was necessary for the classifier to generalize the variability in movement trajectories and grip patterns observed in participants with stroke. Despite the reduction in accuracies, the use of a training set that was 50% the size of the testing set resulted in 91.7% (σ = 3.9%) with participants with stroke, and 95.6% (σ = 1.6%) with healthy participants using the RBF-SVM (Figure 5); and this met our second feasibility criteria. These promising results are indicative of the capabilities of FMG-based sensing systems for grasp detection in individuals with stroke, with minimal setup, in controlled environments.

It is noteworthy that the grasp detection accuracy obtained for this preliminary experimental protocol, corresponds to the detection of grasping of a single object of constant weight and shape. While the use of a variety of movement trajectories, wrist, elbow and shoulder postures, and grasp types introduced variability within the data, this study did not investigate the effect of the weight of the object on FMG grasp detection accuracy. Furthermore, this study also did not seek to establish the effect of removing and redonning the force-sensing band at different positions, and with different levels of tightness, as would be expected in daily use. These variations in object weight, band donning position and band tightness could introduce variability in the FMG signal acquired and impact grasp detection accuracy. It is also possible that other sensing modalities, such as sEMG or MMG may prove more repeatable, and less susceptible to these additional sources of variability when compared to FMG. Based on the promising results obtained in this preliminary investigation, future work should investigate the impact of the aforementioned variables on FMG-based grasp detection accuracy.

## Future Work

The scope of this study is limited to establishing the preliminary feasibility of classifying the FMG signal for grasp detection with participants with stroke. The study evaluated grasp detection accuracy with a single object, of constant weight, in a controlled environment. Future work should explore robustness of FMG signal-processing and feature-extraction techniques for differing objects, various grasp types and movement trajectories in an uncontrolled environment. In addition, FMG-based grasp detection with participants with stroke with moderate to severe impairments should also be evaluated. Furthermore, robust FMG sensing bands and systems will also need to be developed to be practically deployable for grasp detection in uncontrolled environments. This includes an analysis and selection of various force-sensing elements, band backing materials, and battery-based power management systems to make the band wireless. Furthermore, the impact of removal and redonning of the FMG sensing band should be investigated and minimized. Finally, the efficacy of FMG-based grasp detection systems for encouraging grasping and functional activity, as part of daily living, in individuals with stroke who are actively undergoing rehabilitation should also be assessed.

## Conclusion

This study investigated the feasibility of classifying FMG for grasp detection, in individuals with stroke, with a data set that included variations in upper-extremity movement and postures, as would be expected in daily living. FMG classification accuracy was lower and required more training data to achieve the target 90% accuracy for participants with stroke when compared to healthy participants. Despite the noted considerations, FMG-based sensing achieved a high accuracy of approximately 92% for detecting grasping in the presence of other upper-extremity movements with participants with stroke. In addition, a training set size that was 50% of the testing set size was sufficient to achieve approximately 91.5% grasp detection accuracy with participants with stroke. These promising results indicate that FMG sensing may be feasible for monitoring grasping in the presence of upper-extremity movements, in individuals with stroke and mild to moderate upper-extremity impairment.

## Ethics Statement

The study received ethics approval from the SFU and UBC Research Ethics Boards. All participants provided informed consent for their participation in the study.

## Author Contributions

GS designed the experimental device and experimental protocol, implemented data collection software, performed experiments, analyzed experimental results, and participated in manuscript preparation. XJ performed experiments, analyzed experimental results, and participated in manuscript preparation. LS facilitated participant enrollment, administered functional tests, and participated in manuscript preparation. JE contributed to discussions, analysis, and participated in manuscript revisions. CM supervised the project, contributed to discussions, analysis, and participated in manuscript revisions. All the authors read and approved the submitted manuscript.

## Conflict of Interest Statement

The principal investigator, CM, and members of his research team have a vested interest in commercializing the technology tested in this study, if it is proven to be successful and may benefit financially from its potential commercialization. The data are readily available upon request.

## Abbreviations

ADL, activities of daily living; FMG, force myography; FN, false negative; FP, false positive; FSR, force-sensitive resistor; LDA, linear discriminant analysis; MMG, mechanomyography; RBF, radial basis function; SVM, support vector machine; TN, true negative; TP, true positive.
